# Supplementary material for: From sequence to enzyme mechanism using multi-label machine learning
Source: BMC Bioinformatics. 2014 May 19;15:150. doi: 10.1186/1471-2105-15-150 (PMC4229970; doi:10.1186/1471-2105-15-150)
Supplement: Additional file 2 — Java code of ml2db. Additional file ml2db_code.tar.gz contains the Java source code to run the multi-label machine learning experiments and save the results to database. The code’s Javadoc is included. [file 1471-2105-15-150-S2.zip › additional file 2/ml2db/ml2db/doc/main/ml2db/mulan/mulanarff/NeighboursPrinterTest.html]

NeighboursPrinterTest


JavaScript is disabled on your browser.


- Overview
- Package
- Class
- Use
- Tree
- Deprecated
- Index
- Help

- Prev Class
- Next Class

- Frames
- No Frames

- All Classes

- Summary:
- Nested |
- Field |
- Constr |
- Method

- Detail:
- Field |
- Constr |
- Method


main.ml2db.mulan.mulanarff

## Class NeighboursPrinterTest

- java.lang.Object
- - main.ml2db.mulan.mulanarff.NeighboursPrinterTest

- ---

    

  ```
  public class NeighboursPrinterTest
  extends java.lang.Object
  ```

  Class

  Version:
  :   5 Oct 2012

  Author:
  :   Luna De Ferrari ldf(at)st-andrews.ac.uk or
      luna.deferrari@st-andrews.ac.uk

- - ### Constructor Summary

    Constructors

    Constructor and Description

    `NeighboursPrinterTest()`
  - ### Method Summary

    Methods

    | Modifier and Type | Method and Description |
    | --- | --- |
    | `static NeighboursPrinter` | `getTestNeighboursPrinter()` PRINTING ONLY ! . |
    | `static NeighboursPrinter` | `getTrainNeighboursPrinter()` |
    | `void` | `testGetTestInstanceAndNeighboursMap()` Test method for `NeighboursPrinter.getInstanceAndNeighboursMap()` . |
    | `void` | `testGetTrainInstanceAndNeighboursMap()` Test method for `NeighboursPrinter.getInstanceAndNeighboursMap()` . |

    - ### Methods inherited from class java.lang.Object

      `equals, getClass, hashCode, notify, notifyAll, toString, wait, wait, wait`

- - ### Constructor Detail


    - #### NeighboursPrinterTest

      ```
      public NeighboursPrinterTest()
      ```
  - ### Method Detail


    - #### testGetTestInstanceAndNeighboursMap

      ```
      public void testGetTestInstanceAndNeighboursMap()
      ```

      Test method for
      `NeighboursPrinter.getInstanceAndNeighboursMap()`
      .


    - #### testGetTrainInstanceAndNeighboursMap

      ```
      public void testGetTrainInstanceAndNeighboursMap()
      ```

      Test method for
      `NeighboursPrinter.getInstanceAndNeighboursMap()`
      .


    - #### getTestNeighboursPrinter

      ```
      public static NeighboursPrinter getTestNeighboursPrinter()
      ```

      PRINTING ONLY ! .


    - #### getTrainNeighboursPrinter

      ```
      public static NeighboursPrinter getTrainNeighboursPrinter()
      ```


- Overview
- Package
- Class
- Use
- Tree
- Deprecated
- Index
- Help

- Prev Class
- Next Class

- Frames
- No Frames

- All Classes

- Summary:
- Nested |
- Field |
- Constr |
- Method

- Detail:
- Field |
- Constr |
- Method
